# Retinal Development in Infants and Young Children with Achromatopsia

**DOI:** 10.1016/j.ophtha.2015.03.033

**Published:** 2015-10

**Authors:** Helena Lee, Ravi Purohit, Viral Sheth, Rebecca J. McLean, Susanne Kohl, Bart P. Leroy, Venki Sundaram, Michel Michaelides, Frank A. Proudlock, Irene Gottlob

**Affiliations:** 1 The University of Leicester Ulverscroft Eye Unit, Robert Kilpatrick Clinical Sciences Building, Leicester Royal Infirmary, Leicester, UK; 2 Molecular Genetics Laboratory, Institute for Ophthalmic Research, Department of Ophthalmology, Tuebingen, Germany; 3 Department of Ophthalmology & Center for Medical Genetics Ghent University Hospital & Ghent University De Pintelaan, Ghent, Belgium; 4 UCL Institute of Ophthalmology and Moorfields Eye Hospital, London, UK

Normally, postnatal development of the human retina involves centrifugal displacement of the inner retinal layers (IRLs) from the fovea, centripetal migration of the cone photoreceptors into the fovea, and elongation of the photoreceptors with age.^1,2^ It is not clear whether retinal development in infants and young children with achromatopsia (ACHM) occurs in a similar way and whether any retinal changes that occur are progressive in early childhood.

To answer this question, we undertook a longitudinal optical coherence tomography (OCT) study of in vivo foveal development in a cohort of 10 children with a confirmed genetic diagnosis of ACHM (Table 1, available at www.aaojournal.org). The study adhered to the tenets of the Declaration of Helsinki and was approved by the local ethic committee. Volumetric foveal scans of the foveal region were obtained using the Bioptigen handheld spectral domain OCT as previously described.^3^ Each retinal layer was segmented using ImageJ software (available at: www.rsbweb.nih.gov/ij; accessed May 11, 2012). Longitudinal data were available for 7 of the 10 children with ACHM, with a mean follow-up time of 18.9 months (range, 5.3–35.5). A total of 55 cross-sectional (n = 6) and longitudinal (n = 49) examinations were obtained. These were compared with 110 cross-sectional examinations obtained from 59 age-, gender-, and race-matched controls. The mean age at the time of examination was 40.6 months (range, 2.4–98.7) for the ACHM group and 40.6 months (range, 1.4–120.9) for the control group.

In all of the participants with ACHM, there was evidence of foveal hypoplasia (presence of the normally absent IRLs at the fovea) at each visit on OCT examination. A delay in migration of the photoreceptors into the central fovea was evident in the youngest ACHM participants. There was evidence of photoreceptor disruption (consisting of ellipsoid [ISe] disruption and/or a hyporeflective zone) in the ACHM group, the severity of which was graded using a modified version of a previously described grading system^4^ (Fig 1; Table 1, available at www.aaojournal.org). We assessed for changes in the grade of photoreceptor disruption in 14 eyes of 7 participants for whom longitudinal data were available and observed increases (5 eyes), decreases (1 eye), and no change (3 eyes) in the grade of photoreceptor disruption over the follow-up period. Interestingly, in 5 eyes both an increase and decrease in the grade of photoreceptor disruption was observed at different time points during the study, which may represent dynamic alterations in the balance between ongoing retinal development and changes in photoreceptor cell integrity in children with ACHM. An alternative explanation is that the differences are artifactual, because exactly the same foveal cut may not have been obtained on each longitudinal visit owing to a combination of continuously altering biometric parameters of the pediatric eye and variations in positioning of both the child and examiner during scan acquisition between visits.

To define the differences between the ACHM and control groups with regard to retinal layer thickness measurements and their rate of change over time, linear mixed modelling was performed using STATA (Copyright 1996–2014, StataCorp). Overall retinal thickness measurements and rate of increase in retinal thickness with age were reduced significantly at the fovea, parafovea (500 μm from the central fovea), and perifovea (1500 μm from the central fovea) in ACHM (Fig 2A).

The foveal IRLs were 5 times thicker in ACHM than mean control values (*P* < 0.0001), as a result of significantly increased thicknesses of the ganglion cell (GCL), inner plexiform, inner nuclear (INL), and outer plexiform (OPL) layers. There was an age-related decrease in foveal IRL thickness in ACHM, owing to regression of the GCL, INL, and OPL (Fig 2B).

In contrast, the foveal outer retinal layers (ORLs) in ACHM were significantly thinner, being 0.6 times thinner than mean control values (*P* < 0.0001), which was attributable to reductions in the photoreceptor inner segment (IS), outer segment (OS), and outer nuclear layer (Fig 2C). Foveal IS and OS measurements in ACHM show a bimodal distribution as a result of the presence of ISe disruption and/or a hyporeflective zone preventing accurate measurements of the IS and OS in a number of cases (n = 30; Fig 2F). There was no intergroup difference in the rate of increase in foveal ORL thickness with age. Mixed linear regression analysis showed a significant negative correlation between the thickness of the ORLs and the thickness of the IRLs at the fovea (β = −0.52; *P* < 0.0001), which suggests that cone photoreceptors influence IRL migration during retinal development.

The perifoveal IRLs were significantly thinner in ACHM (*P* < 0.01; Fig 2B), which was attributable to changes specifically in the plexiform layers (Fig 2D, E). It is likely that the lack of input from normal, functional cone photoreceptors triggers remodeling and alterations in downstream intraretinal connectivity.

The parafoveal ORLs were uniformly thinner in ACHM at all ages (*P* < 0.0001), owing to reductions in IS, OS, and retinal pigment epithelium (RPE) measurements. Perifoveal ORL thickness in ACHM did not differ from controls at birth. However, after 12 months of age a significant intergroup difference emerged, with thinner perifoveal ORLs in ACHM (Fig 2C). This difference can be attributed to increasing ORL thickness with age in the controls, whereas the reverse occurred in ACHM. A contrasting pattern of changes in the RPE takes place across all measured retinal locations, where the RPE becomes thinner with age in ACHM and thicker with age in controls.

We have shown that retinal development is not arrested in children with ACHM, but is ongoing albeit at a reduced rate and magnitude in comparison with controls, with consequences for all retinal layers. We suggest that ACHM is a continuously altering and progressive process in the developing retina. With human gene therapy trials imminent,^5^ our results suggest that therapy should be considered at early ages, while the photoreceptors are still developing, and thereby potentially facilitating normal retinal maturation.

## Figures and Tables

**Figure 2:**
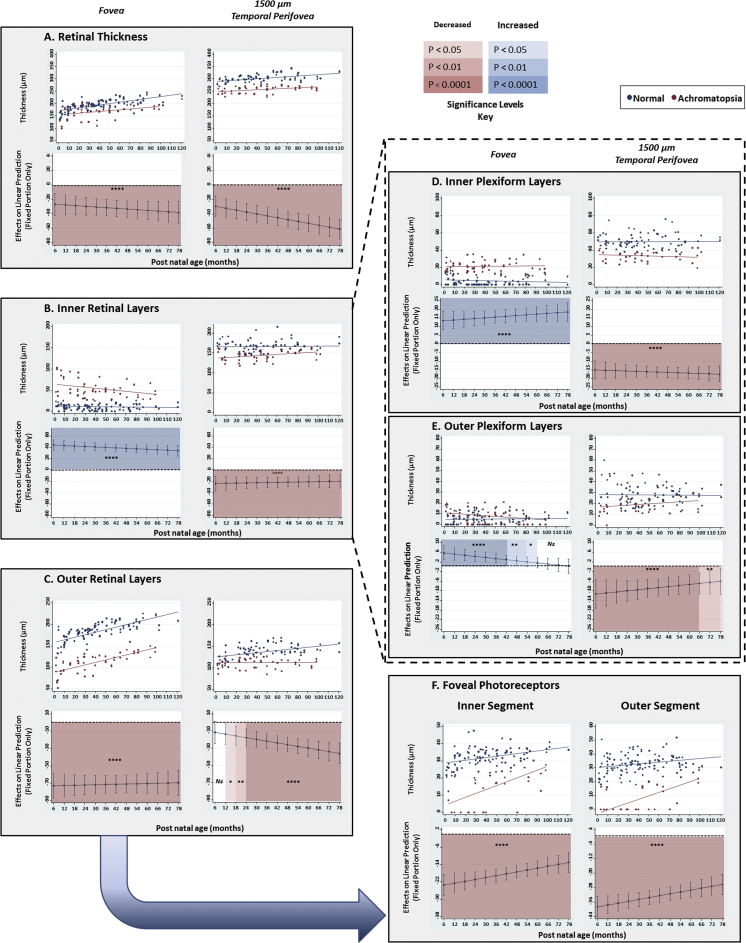
Examples of the developmental trajectories for the retinal thickness (RT), inner retinal layers (IRLs), outer retinal layers (ORLs), inner plexiform layer (IPL), and outer plexiform layer (OPL) at the fovea and temporal perifovea and the photoreceptor inner segment (IS) and photoreceptor outer segment (OS) at the fovea. The upper plots for each panel show the trajectories plotted over a time period spanning 0–120 months postnatal age. Each point represents a single value from each optical coherence tomography (OCT) examination. The lines of best fit (trend lines) are shown in red and blue for the achromatopsia (ACHM) and control groups, respectively. The lower plots for each panel represent the difference between the best fit lines for ACHM and control groups (in the upper plots) with the error bars representing the 95% confidence intervals. By calculating partial derivatives of the interaction term the significant differences between ACHM and control groups were estimated at 13 specified time points, namely, 6, 12, 18, 24, 36, 42, 48, 54, 60, 66, 72, and 78 months postnatal age. Red colors indicate where there is a significantly thinner retinal layer in ACHM and blue colors a significantly thicker retinal layer in ACHM with increasing depth of color representing the level of significance.
