# Supplementary material for: Retinal Development in Infants and Young Children with Achromatopsia
Source: Ophthalmology. 2015 Oct;122(10):2145–7. doi: 10.1016/j.ophtha.2015.03.033 (PMC4582068; doi:10.1016/j.ophtha.2015.03.033)
Supplement: Figure 1 [file mmc1.pdf]

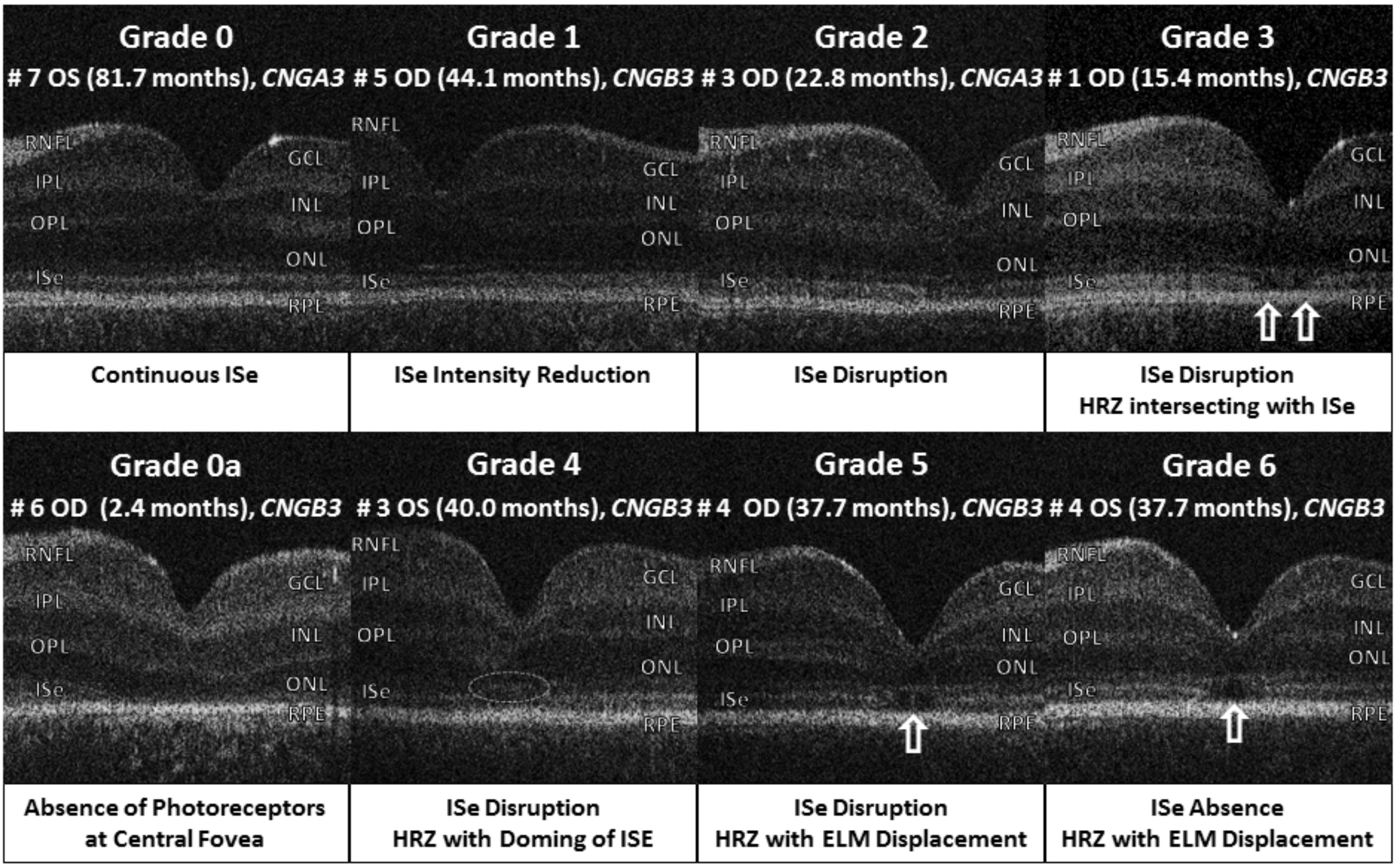

Figure 1: Representative examples of optical coherence tomography scans illustrating the different grades of severity of photoreceptor disruption.

Participants were graded into one of seven categories: (0) Continuous ISe, (1) ISe intensity reduction, (2) ISe disruption with no visible HRZ, (3) ISe disruption with HRZ intersecting with the ISE, (4) ISe disruption with HRZ causing displacement of the ISe (white arrows), (5) ISe disruption with HRZ causing displacement of the ELM (white arrow), (6) ISe absence with ELM displacement (white arrow) or (7) Outer retinal atrophy (Not Shown). A grade of 0a was given to very young infants in whom the central foveal photoreceptors had not yet developed and therefore could not be graded for severity of photoreceptor disruption.

ISe = ellipsoid; HRZ = hypo-reflective zone; ELM = external limiting membrane
