# Supplementary material for: Retinal Development in Infants and Young Children with Achromatopsia
Source: Ophthalmology. 2015 Oct;122(10):2145–7. doi: 10.1016/j.ophtha.2015.03.033 (PMC4582068; doi:10.1016/j.ophtha.2015.03.033)
Supplement: Table 1 [file mmc2.pdf]

Table 1: Clinical and Genetic Characteristics of Achromatopsia Participants

			BCVA (LogMAR)		Photoreceptor Disruption Grade		Refraction			Allele 1		Allele 2		
ID	Sex	Age at Each Visit (months)	Right Eye	Left Eye	Right Eye	Left Eye	Right Eye	Left Eye	Gene	Nucelotide Alteration	Polypeptide Alteration	Nucelotide Alteration	Polypeptide Alteration	ERG
1*	F	15.4	1.6	1.6	3	3	+7.50/-2.00@ 11	+6.00/-1.50@ 161	*CNGB3*	c.1148delC	p.T383IfsX13	c.1148delC	p.T383IfsX13	Absent Cone Response
2*	M	70.8	0.9	0.9	4	3	+4.00/-2.00@ 14	+4.00/-2.00@ 171	*CNGB3*	c.1148delC	p.T383IfsX13	c.1148delC	p.T383IfsX13	Absent Cone Response
		78.0	1.2	1.0	5	2	+4.00/-2.00@ 14	+4.00/-2.00@ 171						
3	M	22.8	0.9	0.9	2	3	+5.00	+5.00	*CNGA3*	c.661C>T	p.R221X	c.1768G>A	p.E590K	Severe Generalised Cone Dysfunction
		28.5	1.2	1.2	3	4	+5.00	+5.00						
		40.0	0.4	0.4	3	4	+2.00/+1.50@ 90	+2.00/+1.50@ 90						
4	F	22.4	2.0	----	3	3	+4.50	----	*CNGB3*	c.1148delC	p.T383IfsX13	c.1148delC	p.T383IfsX13	Unreliable
		37.7	1.05	1.5	4	NA	+1.75/-0.75@ 10	+0.75						
5	M	37.4	0.9	0.9	5	6	+6.00/-5.00@ 175	+6.00	*CNGB3*	c.1148delC	p.T383IfsX13	c.1148delC	p.T383IfsX13	Severe Generalised Cone Dysfunction
		44.1	1.3	1.3	6	6	+6.00/-5.00@ 175	+6.00						
		50.2	1	1	2	2	+6.00/-5.00@ 175	+6.00						
		58.3	1	1	1	1	+6.00/-5.00@ 175	+6.00						
6	F	2.4	1.5	1.5	1	2	+2.50/-5.00@ 180	+4.00/-0.75@ 180	*CNGB3*	c.1148delC	p.T383IfsX13	c.1148delC	p.T383IfsX13	Severe Generalised Cone Dysfunction
		6.3	1.5	1.5	2	2	+2.50/-5.00@ 180	+4.00/-0.75@ 180						
		9.0	1.3	1.3	1	1	+2.50/-5.00@ 180	+4.00/-0.75@ 180						
		15.2	1.3	1.3	0a	0a	+2.50/-5.00@ 180	+4.00/-0.75@ 180						
		23.3	1.3	1.3	0a	0a	+2.50/-5.00@ 180	+4.00/-0.75@ 180						
7	F	81.7	0.925	0.925	5	5	+4.25/-1.50@ 10	+4.50/-1.75@ 170	*CNGB3*	c.1148delC	p.T383IfsX13	c.1148delC	p.T383IfsX13	Severe Generalised Cone Dysfunction
		93.8	0.65	0.85	6	Ungradable	+4.75/-1.75@ 10	+4.25/-2.00@ 170						
		98.7	0.8	0.725	5	6	+3.75/-2.25@ 12	+3.25/-1.75@ 170						
8	M	50.0	1.3	1.3	5	5	+3.75/-2.00@ 15	+4.25/-2.00@ 0	*CNGB3*	c.1148delC	p.T383IfsX13	c.1148delC	p.T383IfsX13	----
9	F	29..5	2.1	2.1	0	0	+6.75/-1.25@8	+7.00/-1.5@163	*CNGA3*	c.848G>A	p.R283Q	c.1116delC	p.V373	Absent Cone Response
10	F	3.2	F&F	F&F	0	0	----	----	*CNGB3*	c.886_896 delinsT	p.T296YfsX9	c.1397T>A	p.M466k	Absent Cone Response
		8.5	1.5	1.5	0	0	+7.5/-0.75 @170	+8.50						

ID = identification number; M = male; F = female; BCVA = best corrected visual acuity; LogMAR = Logarithm of the Minimum Angle of Resolution; ERG = electroretinogram

* $^{+}$ indicate sibling pairs

---- indicates not assessed or could not be performed
